# Emodin suppresses hepatocellular carcinoma growth by regulating macrophage polarization via microRNA-26a/transforming growth factor beta 1/protein kinase B

**DOI:** 10.1080/21655979.2022.2061295

**Published:** 2022-04-07

**Authors:** Jiao Yin, Xiansheng Zhao, Xuejiao Chen, Guanxin Shen

**Affiliations:** a Department of Immunology, School of Basic Medicine, Hubei University of Arts and Science, 296 Longzhong Road, Xiangyang, 441053, China; b Department of Hepatology, Ningbo Huamei Hospital, University of Chinese Academy of Sciences, 175 Yongfeng Road, Ningbo, 315010, China; c Department of Immunology, School of Basic Medicine, Tongji Medical College, Huazhong University of Science and Technology, 13 Hangkong Road, Wuhan, 430030, China

**Keywords:** Hepatocellular carcinoma, emodin, macrophage polarization, TGF-β1, miR-26

## Abstract

Accumulating evidence has demonstrated that M2 macrophages contribute to the progression of hepatocellular carcinoma (HCC). Emodin is an anti-tumor agent and potentially regulates macrophage polarization. This study aims to explore the effect of emodin on M2 polarization in HCC and its underlying mechanism. After co-culture systems of M2 macrophages and HCC (HepG2 and Huh7) cells were established, it was shown that co-culture with M2 macrophages could promote both the proliferation and invasion of HepG2 and Huh7 cells. Emodin induces the transformation of M2 to M1 macrophages, thereby inhibiting the proliferation and invasion of HepG2 and Huh7 cells mediated by co-culturing with M2 macrophages. Based on bioinformatics analysis and in vitro validation, it was found that the effect of emodin on M2 polarization was regulated by the microRNA-26a (miR-26)/Transforming growth factor beta 1 (TGF-β1)/Protein kinase B (Akt) axis. *In vivo* analysis showed that co-culturing with M2 macrophages markedly facilitated the growth of HepG2 cells, which was significantly inhibited by emodin. Western blot analysis on xenografts confirmed that emodin could induce transformation of M2 to M1 macrophages and reverse the up-regulation of PCNA, TGF-β1, and p-Akt induced by M2 macrophages. In summary, our findings uncover a novel mechanism behind the anti-tumor effects of emodin that regulates M2 polarization via miR-26a/TGF-β1/Akt to suppress HCC growth.

## Graphical abstract

**Figure:**
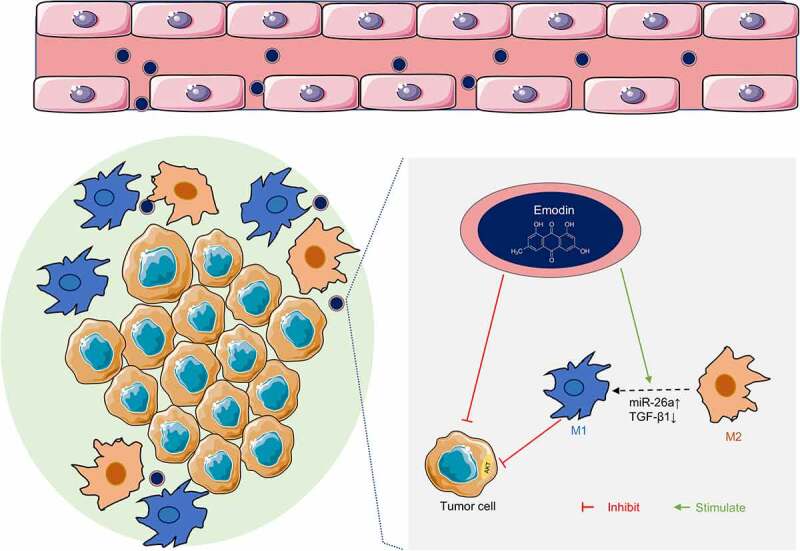

## Introduction

As one of the most prevalent malignancies, liver cancer remains the main cause of tumor-related deaths worldwide. Statistics in 2015 reported that the incidence of liver cancer ranks the 4th among malignancies, and live cancer ranks the 3rd in causes of cancer-related deaths, the 5-year overall survival rate of which is rather low in China [1]. Of all cases of liver cancer, more than 90% are diagnosed as hepatocellular carcinoma (HCC). The treatments for HCC mainly include surgical resection, radiotherapy, chemotherapy, and liver transplantation [2]. Despite the advances in the management of HCC in the last few decades, the prognosis of HCC patients remains unsatisfactory. According to the research on murine and human tumors, immune system has been proven to play a critical role in recognizing and eliminating transformed malignant cells [3,4]. In recent years, cancer immunotherapy has gradually emerged as one of the most promising therapies to improve patient survival [5].

It is well known that tumor microenvironment (TME) is crucial for the progression of malignancies, which can effectively prevent the body’s immune system from attacking tumors [6]. As the main component of TME, tumor-associated macrophages (TAMs) include classically activated macrophages (M1) with anti-tumor and pro-inflammatory properties and alternatively activated macrophages (M2) with immunosuppressive, pro-tumor and anti-inflammatory effects, which play important roles in promoting tumor development or anti-cancer behaviors in different environments [7]. During the progression of tumors, TAMs are mainly polarized toward M2 macrophages [8]. Accumulating evidence has demonstrated that M2 macrophages play a key role in tumorigenesis, growth, migration, and formation of vascular and lymphatic vessels, which hence are potent targets for cancer immunotherapy [9]. A clinical study has demonstrated that the increase in the number of M2 macrophages is highly correlated with tumor metastasis, angiogenesis, and poor prognosis of papillary thyroid carcinoma [10]. In addition, 11,revealed that M2 macrophages contribute to poor prognosis, increased tumor nodules, and venous infiltration in HCC patients. It is well recognized that the polarization of TAM is not the ultimate state, but a reversible dynamic process, which may change over time and is affected by the microenvironment in different physiological and pathological processes [12]. Hence, it is necessary to explore small-molecule drugs that can reverse M2 macrophages into anti-tumor M1 macrophages and inhibit tumor growth and metastasis to facilitate development of cancer immunotherapy in the future.

Emodin is a natural anthraquinone derived from the root of *Rheum palmatum L*, which has shown significant anticancer activities in various cancers both *in vivo* and *in vitro* [13–15], whereas its underlying anticancer mechanisms are largely unclear. Emodin represses the excessive response of both M1 and M2 polarization, and therefore plays a role in restoring macrophage homeostasis in multiple pathologies [16]. 17,demonstrated that emodin could effectively ameliorate asthmatic airway inflammation and decrease M2 polarization *in vivo*. A previous study by 18,has shown that emodin suppresses breast cancer growth by inhibiting M2 polarization. The anti-tumor effect of emodin on HCC has been reported in many studies; however, whether emodin could regulate the polarization of M2 macrophages in HCC has seldom been studied.

Based on the aforementioned literature, we wonder whether emodin can induce the transformation of M2 to M1 macrophages to perform the anti-cancer effect on HCC. Therefore, the present study aimed to investigate the effect of emodin on the M2 polarization in HCC, and preliminarily explore the molecular mechanism. It is expected that emodin harbors the potential for the treatment of HCC as a new effective agent.

## Materials and methods

### Cell culture and treatment

Human HCC cell lines HepG2 and Huh7 were purchased from ATCC (Manassas, USA) and JCRB (Osaka, Japan), respectively. Human leukemia monocytic cell-line THP-1 was obtained from Shanghai Cell Biological Institute of the Chinese Academy of Science (Shanghai, China). HepG2 and Huh7 cells were maintained in DMEM while THP-1 cells in RPMI 1640, all of which contained 10% FBS and 100 U/mL of penicillin/streptomycin.

THP-1 cells were incubated with 150 nM PMA (cat. P8139, Sigma-Aldrich, USA) for 24 h to differentiate into M0 macrophages, followed by 24-hour incubation with 20 ng/mL of IL-4/IL-13 for M2 polarization to obtain M2 macrophages [19].

Transwell inserted with a 0.4 μm porous membrane (Corning, NY, USA) to separate the upper and lower chambers was applied in the co-culture experiments [20]. Briefly, two types of cells were, respectively, seeded into the upper and lower chambers for adherence. After the cell adhesion, the upper chamber with M2 macrophages was placed directly on the lower chamber containing HepG2 or Huh7 cells, and co-cultured in serum-free RPMI 1640 for 24 h. HepG2 cells, Huh7 cells, and M2 macrophages alone were also cultivated for 24 h in the same medium as controls.

The stock solution of emodin (1 mM, Sigma-Aldrich) was prepared with DMSO and stored at −20°C in the dark before use. Recombinant human Transforming growth factor beta 1 (TGF-β1) protein (cat. ab50036, Abcam, USA) was utilized with a concentration of 5 ng/mL for 24 h. TGF-β1 monoclonal antibody (TGF-β1Ab; cat.69012-1-Ig, Proteintech, USA) for neutralizing TGF-β1 was used 1 h prior to other treatment.

## Enzyme-linked immunosorbent assay (ELISA)

Conditioned medium was respectively harvested from M2 (the monoculture of M2 macrophages), HepG2 (the monoculture of HepG2 cells), Huh7 (the monoculture of Huh7 cells), and co‑culture (the co-culture of M2 macrophages and HepG2 or Huh7 cells) groups for centrifugation (1,200 × g, 5 min) at 4°C. Subsequently, TGF-β1 levels in the conditioned medium were quantified using Human TGF-β1 ELISA kits [21] (cat. ab100647, Abcam) according to the manufacturer’s instructions.

## RT-qPCR

After the incubation, cells were harvested to extract the total RNA using the TRIzol reagent. Then, the collected total RNA was reversely transcribed into cDNA using Transcriptor first-strand cDNA synthesis kit (cat.4379012001, Roche, Germany) with the primer of studied RNAs. The RNA expression levels were determined by qPCR on a 7500 Real-Time PCR System (Applied Biosystems Inc, USA). GAPDH and U6 served as the internal reference to, respectively, normalize the expression levels of mRNA (TGF-β1, Arg-1, IL-10, iNOS, and TNF-α) and miRNA (miR-320a, miR-125a, miR-26a, and miR-21). Relative quantitative analysis was performed based on the 2^−ΔΔCt^ method [22]. Primer sequences of the studied RNAs were listed as follows: GAPDH (forward (F): 5’- GCAACTAGGATGGTGTGGCT-3’; reverse (R): 5’-TCCCATTCCCCAGCTCTCATA-3’), TGF-β1 (F: 5’- AAATGAAGGGAGGCGATCAGG-3’; R: 5’-AATTGGTGCCACATGGCTTG-3’), Arginase-1, (Arg-1, F: 5’- ACCTGAAACCAAGTCCCAGC-3’; R: 5’- CGAGCAAGTCCGAAACAAGC-3’), IL-10 (F: 5’- ATGCGGTCTTTTTGATGCCC-3’; R: 5’-ATAAGATCCTGCTGGCGCTC-3’), iNOS (F: 5’-GAACAGGGCTTCTCAGTGGG-3’; R: 5’- ACCCCTGTTTCAACGACCTC-3’), TNF-α (F: 5’- GAGACAGATGTGGGGTGTGAG-3’; R: 5’- TCCTAGCCCTCCAAGTTCCA-3’), U6 (F: 5’-CTCGCTTCGGCAGCACA-3’; R: 5’-AACGCTTCACGAATTTGGGAAT-3’), miR-320a (F: 5’-AAGGGATCGCGGGCG-3’; R: 5’-TGCGTGTCGTGGAGTC-3’), miR-125a (F: 5’-GCTCCCTGAGACCCT-3’; R: 5’-GAGCAGGCTGGAGAA-3’), miR-26a (F: 5’-ACACTCCAGCTGGGTTCAAGTAATCCAGGATAGGC-3’; R: 5’-CTCAACTGGTGTCGTGGA-3’), miR-21 (F: 5’-TACCTCGAGTGTCTGCTTGTTTTGCCT-3’; R: 5’-TACGAATTCTGTTTAAATGAGAACATT-3’).

## 5-Ethynyl-2’-deoxyuridine (EdU) incorporation assay

After the treatment according to the study design, cells of different groups were incubated with 1 μM EdU (Life, China) for 2 h and fixed in 4% polyformaldehyde (PFA) for 1 h. The cells were subjected to permeabilization with 0.5% Triton X-100 for 15 min before reaction with Apollo dye reaction liquid for 40 min. The nuclei were counterstained with DAPI and photographed under a fluorescence microscope. The ratio of EdU positive cells was finally calculated to evaluate cell proliferation [23].

## Transwell assay

Transwell chamber containing Matrigel (Corning) was utilized to investigate invasive cells *in vitro* [24]. In brief, 200 μL cell suspension was added in upper chambers. In the lower wells, the fresh medium was supplemented with 5% FBS as a chemo-attractant. After 24-hour incubation, cells passing through the filter were fixed and stained with 0.1% crystal violet. They were observed under an optical inverted microscope. Five random fields were selected for counting and the average was taken.

## CCK-8 assay

To investigate the effect of emodin on the cell viability of M2 macrophages, Huh7 cells, and HepG2 cells, cells were treated with a series of concentrations of emodin (0, 25, 50, 100, and 200 μM, respectively) for 24 h. Cell viability was assessed by Cell Counting Kit-8 (CCK-8) assay [25]. After 24-hour cultivation, the medium was replaced with 10% CCK-8 reagent (Dojindo, Japan) in each well, and the plates were incubated for another 1.5 h. Finally, the absorbance of each well at 450 nm was detected with a microplate reader (BioTek, USA).

## Flow cytometry (FCM)

FCM was performed to determine the abundance of M1/2 macrophages based on M2-specific marker CD206 and M1-specific marker CD86 [26]. After being washed three times in PBS, the macrophages with different treatments were stained with FITC-conjugated CD68 (cat. ab134351, Abcam) or PE-conjugated CD206 (cat.FAB25342P, R&D Systems, USA) antibody for 30 min at 25°C. Finally, after cells were washed twice again, flow cytometric analysis was performed on a FACSCalibur flow cytometer (BD Biosciences, USA), and the results were analyzed with FlowJo software.

## miRNA mimic and inhibitor transfection

As previously described [27], the overexpression and suppression of miR-26a inhibitors in 293 T or M2 macrophages were performed by transfecting miR-26a mimics and inhibitors, respectively. The non-targeting miRNA mimics or inhibitor controls (NC mimics or NC inhibitors) were also transfected as a negative control for miR-26a overexpression or suppression. The verification of the transfection efficiency of miR-26a mimics and inhibitors was conducted by performing RT-qPCR as described above.

## Luciferase reporter assay

Using the online bioinformatics tool TargetScan (http://www.targetscan.org/) [28], the target gene of miR‑26a and TGF-β1, and the corresponding target site were predicted. The sequence of the miR‑26a wild‑type binding site on TGF-β1 (TGF-β1 WT) or the binding site mutant sequence (TGF-β1 MUT) was inserted into pmirGLO vectors (GenePharma, China). The 293 T cells were co-transfected with pmirGLO-TGF-β1 WT or pmirGLO-TGF-β1 MUT and miR‑26a mimics or controls. Luciferase activity was assessed 48 h after transfection using a dual-luciferase reporter gene assay kit (cat. E1910, Promega, USA) [29].

## Western blot

The protein expression was detected by Western blot analysis [30]. In brief, the total protein extracted from different groups of cells and tumor tissues was separated by using SDS-PAGE, and then transferred to PVDF membranes, before blotted with specific primary and secondary antibodies. All antibodies used in Western blot were purchased from Abcam, and described as follows: anti-TGF beta 1(TGF-β1) antibody (cat.ab92486), anti-pan-AKT antibody (cat.ab8805), anti-Protein kinase B (AKT) (phospho T308) antibody (cat.ab38449), anti-STAT3 antibody (cat.ab68153), anti-STAT3 (phospho S727) antibody (cat.ab32143), anti-Mannose Receptor (CD206) antibody (cat.ab64693), anti-iNOS antibody (cat.ab178945), anti-PCNA antibody (cat.ab29), anti-GAPDH antibody-Loading Control (cat.ab8245), and Goat Anti-Rabbit IgG H&L (HRP) (cat.ab6721). The protein bands were visualized using an ECL Western Blotting Substrate Kit (cat. ab65623, Abcam).

## *In vivo* assay

The experimental processes involving animals were carried out as Zhao et al. reported [20], which were approved by the Ethics review committee of School of Basic Medicine, Tongji Medical College, Huazhong University of Science and Technology. BALB/c nude mice (Guangdong Medical Laboratory Center, China; 4–6 weeks) were randomly divided into three groups (N = 5/per group): HepG2, HepG2/M2, and HepG2/M2+ Emodin groups. Mice in the HepG group were subcutaneously injected with HepG2 cells alone, whereas those in the HepG2/M2, and HepG2/M2+ Emodin groups were subcutaneously injected with HepG2 cells together with M2 macrophages into the back. Mice in the HepG2/M2+ Emodin group received intraperitoneal injection of emodin (dissolved in DMSO; 50 mg/kg/day) [31] after 1 week since inoculation, and those in the other two groups were treated with vehicle. 10 days after cell inoculation, the growth of established xenografts on each mouse was monitored using a Vernier caliper every 5 days (days 10, 15, 20, 25, and 30) and calculated based on the formula: length × width^2^/2. On day 30, mice were sacrificed, and xenografts were harvested for weight and further analysis.

## Statistical analysis

Statistical analysis in this study was performed using GraphPad Prism software. Data were represented as mean ± SD, and analyzed using the student t-test or one-way analysis of variance followed by Tukey’s post hoc test. P < 0.05 indicated that the difference was significant.

## Results

### TGF-β1 secreted by M2 macrophages is involved in the invasion and proliferation of HCC cells

**Figure 1.**
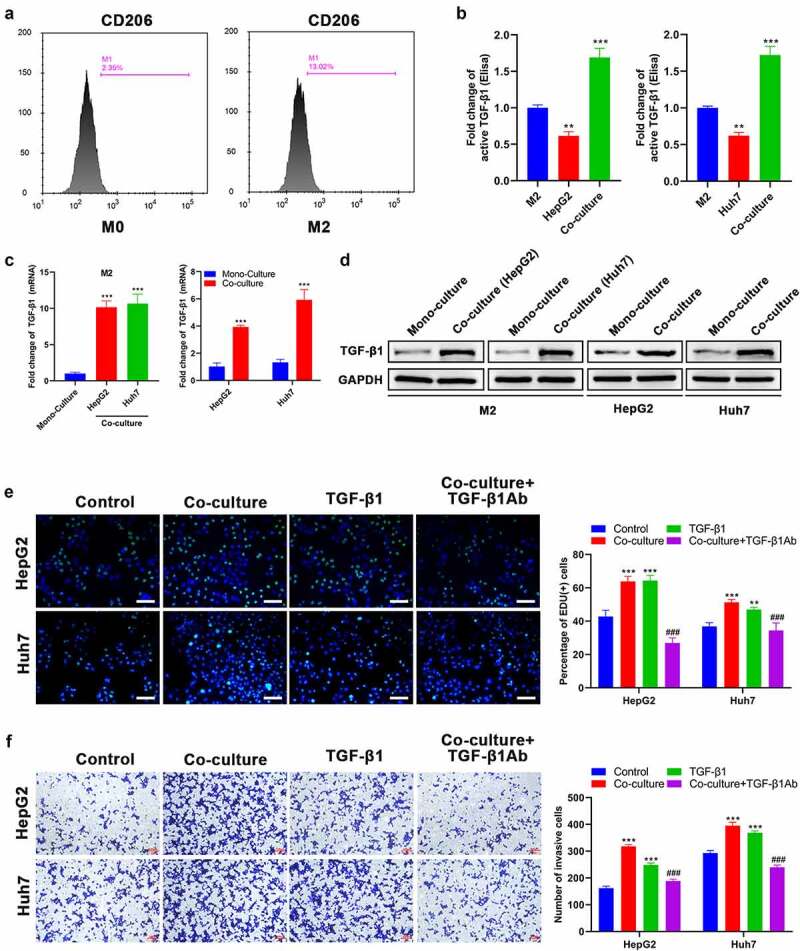
TGF-β1 secreted by M2 macrophages promotes the proliferation and invasion of HCC cells. (a) CD206 expression of THP-1 after treatment with PMA and IL-4/13 was analyzed by FCM. (b) TGF-β1 secretion in mono-culture medium of M2 macrophages and HCC (HepG2 and Huh7) cells and in co-culture medium of M2/HepG2 and M2/Huh7 detected by an ELISA kit. (c) The mRNA and (d) protein expression of TGF-β1 in M2 macrophages, HepG2 cells, and Huh7 cells before and after co-culture was assessed by RT-qPCR and Western blot, respectively. HepG2 and Huh7 cells were cultured with different treatments as follows: mono-culture (used as the control group), co-culture, TGF-β1 treatment, co-culture + TGF-β1Ab pretreatment, and their (e) proliferation and (f) invasion were analyzed using Edu and transwell assays, respectively. **P < 0.01 and ***P < 0.005, vs. M2 or mono-culture or control; ^###^P < 0.005, vs. co-culture. Scale bar = 100 μm.

It has been reported that M2 macrophages are involved in the progression of malignancies by releasing TGF-β1 [32]. Therefore, M2 macrophages were co-cultured with HepG2 or Huh7 cells to investigate the secretion and expression of TGF-β1. Initially, THP-1 cells were treated with PMA to induce the differentiation into M0 macrophages. After further induced by IL-4/IL-13, there was a significant increase in the CD206 expression (Figure 1(a), suggesting that some M0 macrophages differentiated into M2 macrophages. According to the ELISA assay, it was found that the secretion of TGF-β1 from M2 macrophages cultured alone was significantly higher than that from HepG2 and Huh7 cells cultured alone (Figure 1(b). After M2 macrophages were co-cultured with HepG2 or Huh7 cells, the secretion of TGF-β1 was noticeably increased compared with monoculture (Figure 1(b). Then, we analyzed the expression level of TGF-β1 in M2 macrophages, Huh7, and HepG2 cells under conditions of mono-culture and co-culture. The results of both RT-qPCR and Western blot revealed that the TGF-β1 expression level in M2 macrophages from the co-culture group was increased approximately ten times compared with the mono-culture group (Figure 1(c, d). Similarly, the TGF-β1 expression level in HepG2 and Huh7 cells from the co-culture group was also significantly increased compared with the mono-culture group (Figure 1(c, d). Based on the data above, TGF-β1 secretion in co-culture medium was significantly increased and TGF-β1 expression levels of M2 macrophages, HepG2, and Huh7 cells were up-regulated after co-culture. Afterward, Edu incorporation assay revealed that the proliferation ability of both HepG2 and Huh7 cells was remarkably enhanced after co-culture (Figure 1(e). Simultaneously, co-culture with M2 macrophages could also significantly enhance the invasion capacity of HepG2 and Huh7 cells (figure 1f). TGF-β1 treatment showed the same effect on HepG2 and Huh7 cells (Figure 1(e, f). Notably, pre-treatment of TGF-β1Ab before co-culture could inhibit proliferation and invasion of HepG2 and Huh7 cells induced by co-culture (Figure 1(e, f)).

**Emodin suppresses the viability and invasion of HCC cells and induces the transformation of M2 to M1 macrophage**

**Figure 2.**
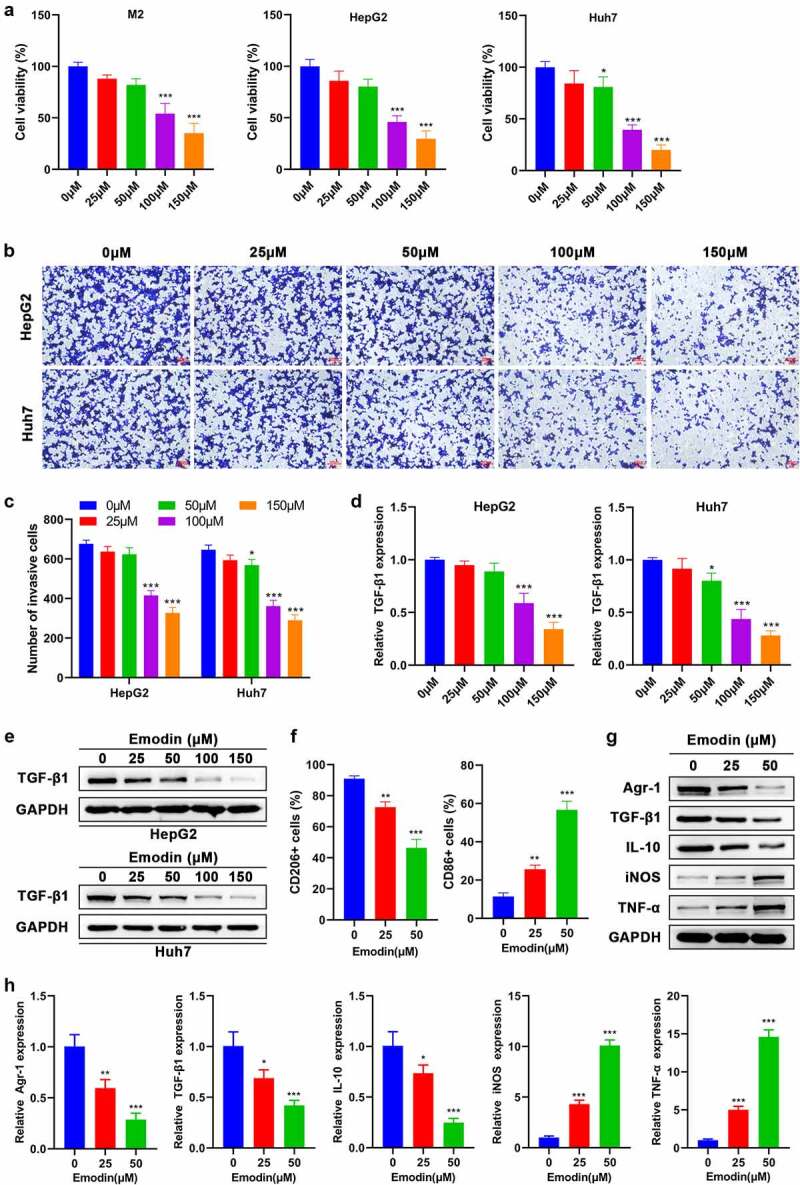
Emodin suppresses the viability of M2 macrophages and HepG2 cells and induces the transformation of M2 to M1 macrophages. (a) Effect of emodin (0, 25, 50, 100, and 150 μM) on the viability of M2 macrophages, HepG2 cells, and Huh7 cells for 24 h. Effect of emodin (0, 25, 50, 100, and 150 μM) on the (b-c) invasion, (d) TGF-β1 mRNA expression, and (e) TGF-β1 protein levels of HepG2 and Huh7 cells for 24 h. M2 macrophages were treated with 0, 25, and 50 μM emodin for 24 h, and (f) CD206 and CD86 expression of M2 macrophages was analyzed by FCM; (g) The protein and (h) mRNA expression of Arg1, TGF-β1, IL-10, iNOS, and TNF-α in M2 macrophages was assessed by Western blot and RT-qPCR, respectively. *P < 0.05, **P < 0.01, and ***P < 0.005, vs. 0 μM.

To investigate the effect of emodin on M2 macrophages, Huh7, and HepG2 cells, they were treated by a series of concentrations of emodin (0–150 μM). The cell viability of M2 macrophages, Huh7 cells, and HepG2 cells was suppressed by emodin (0–150 μM) in a concentration-dependent manner (Figure 2(a). Our data show that the suppressive effect of emodin on the cell viability of both M2 macrophages and HepG2 cells was not significant when the concentration was less than or equal to 50 μM Figure 2(a). Emodin treatment also significantly impaired the invasion of both Huh7 and HepG2 cells once its concentration was higher than 50 μM (Figure 2(b, c)). In addition, emodin treatment caused a decrease of TGF-β1 expression in both Huh7 and HepG2 cells in a concentration-dependent way, as revealed by RT-qPCR and Western blot analysis (Figure 2(d,e)). To investigate whether emodin could stimulate the transformation of M2 macrophages to M1 phenotype, the concentrations of 25 and 50 μM were chosen for the subsequent experiments in order to exclude the obvious suppressive effect of emodin. M2 macrophages were harvested after treated with emodin (0, 25, and 50 μM), and CD206 (M2-specific marker) and CD86 (M1-specific marker) were determined using FCM. It was found that CD206 was reduced, while CD86 was increased significantly, suggesting that emodin potentially changed the phenotype of macrophages from M2 to M1 (figure 2(f). Next, our results further showed that emodin could cause a decrease in the expression of TGF-β1, Arg1, and IL-10 (M2 associated cytokines) and an elevation in the expression of iNOS and TNF-α (M1 associated cytokines) in M2 macrophages at both protein and mRNA levels, confirming that the polarization of the M1 macrophages induced by emodin was accompanied by functional change (Figure 2(g, h)).

## Emodin inhibits the proliferation and invasion of hepG2 cells induced by co-culturing with M2 macrophages

**Figure 3.**
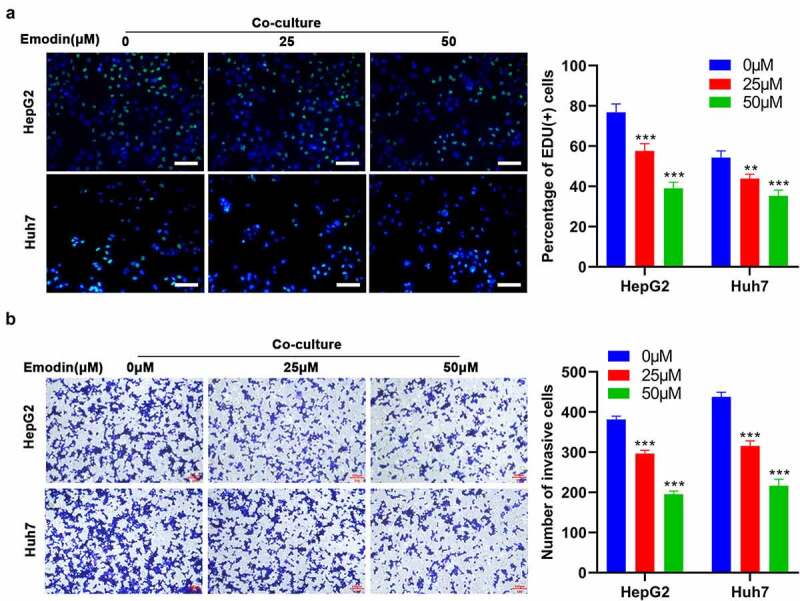
Emodin reverses the effect of promoting the proliferation and invasion of HepG2 cells induced by co-culturing with M2 macrophages. M2 macrophages were treated with 0, 25, and 50 μM emodin, and then co-cultured with HepG2 cells. The (a) proliferation and (b) invasion of HepG2 cells were determined using Edu and transwell assays. *P < 0.05, **P < 0.01, and ***P < 0.005, vs. 0 μM. Scale bar = 100 μm.

To investigate the effect of emodin on the co-culture system of M2 macrophages with HepG2 or Huh7, M2 macrophages were treated with 0, 25, and 50 μM of emodin and then co-cultured with HepG2 or Huh7 cells. As expected, the pretreatment of emodin prior to co-culture significantly reduced both the cell proliferation (Figure 3(a) and invasion (Figure 3(b) of both HepG2 and Huh7 cells in comparison to the control group (0μM). These data collectively suggested that emodin suppressed cell proliferation and invasion of HepG2 and Huh7 cells by inducing polarization of the M1 macrophages in the co-culture system.

## miR-26a/TGF-β1 axis is involved in the polarization of M2 to M1 macrophages induced by emodin

**Figure 4.**
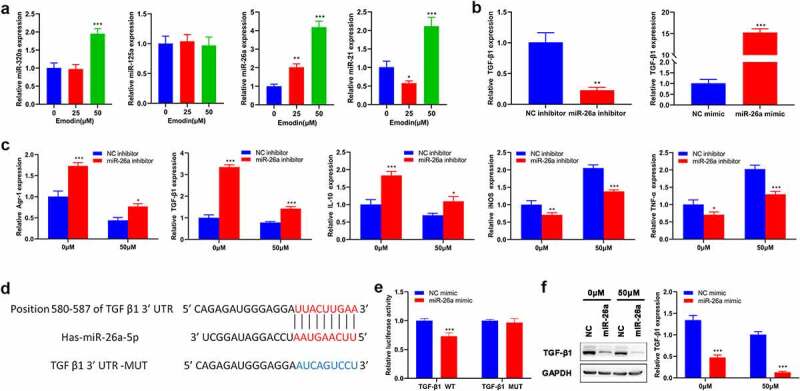
miR-26a contributes to the transformation of M2 to M1 macrophages and directly targets TGF-β1. (a) The expression levels of miR-320a, miR-125a, miR-26a, and miR-21 in M2 macrophages were assessed by RT-qPCR. (b) The expression of miR-26a in M2 macrophages with the transfection of miR-26a mimics, inhibitors, or their corresponding NC. After transfected by miR-26a or NC inhibitors, M2 macrophages were treated with 0 and 50 μM emodin, and (c) the mRNA expression of Arg1, TGF-β1, IL-10, iNOS, and TNF-α in M2 macrophages was assessed by RT-qPCR. (d) The predicted binding sites of miR-26a with TGF-β1. (e) Relative luciferase activity in TGF-β1 wild type and mutant cells with miR-26a mimics was evaluated by dual-luciferase reporter assay. After transfected by miR-26a or NC mimics, M2 macrophages were treated with 0 and 50 μM emodin, and (f) the protein levels of TGF-β1 in M2 macrophages were detected by Western blot. *P < 0.05, **P < 0.01, and ***P < 0.005, vs. 0 μM or NC mimics or NC inhibitors.

Given that microRNAs (miRNAs), including miR-320a [33], miR-125a [34], miR-26a [35], and miR-21 [36], have been implicated in the TAM polarization, the expression of the above-mentioned miRNAs in M2 macrophages with different concentrations of emodin was subsequently detected. RT-qPCR results showed that only the expression levels of miR-26a were consistently elevated with increased emodin concentrations (Figure 4(a)), suggesting that miR-26a might be a mediator in the effect of emodin on the transformation of M2 to M1 macrophages. Since the regulatory role of emodin in macrophage polarization and miR-26a expression at the concentration of 50 μM was more obvious than that of 25 μM, 50 μM was adopted for further analysis. To determine whether miR-26a can regulate macrophage polarization by emodin, miR-26 inhibitors, miR-26a mimics, or their corresponding controls (NC) were transfected into M2 macrophages. The transfection efficiency in M2 macrophages confirmed by RT-qPCR revealed that the transfection of miR-26a inhibitors significantly reduced miR-26a expression, while that of miR-26a mimics noticeably increased miR-26a expression (Figure 4(b)). Interestingly, knockdown of miR-26a caused a significant increase in the expression of TGF-β1, Arg1, and IL-10 in M2 macrophages that were treated with or without emodin (50 μM). On the contrary, the expression of iNOS and TNF-α was significantly inhibited by silencing miR-26a (Figure 4(c)). These data suggested miR-26a might be involved in the polarization of M2 to M1 macrophages induced by emodin.

Since miRNAs generally play biological functions by modulating the transcription of their target genes, the target gene of miR-26a involved in the role of emodin in macrophage polarization was subsequently predicted by using TargetScan. TGF-β1, one of the predicted genes in this study, has been reported to directly bind to miR-26a and participate in macrophage polarization in various cancers. Then, we performed Luciferase reporter assay and it was confirmed that TGF-β1 was a direct target of miR-26a (Figure 4(d,e)). Western blot analysis further validated that the protein expression of TGF-β1 could be directly regulated by miR-26a (figure 4(f)). Collectively, these data suggested that miR-26a might be involved in the polarization of M2 to M1 macrophages induced by emodin via TGF-β1.

**Down-regulated miR-26a blocks the effect of emodin on the proliferation and invasion of HepG2 cells from the co-culture system by suppressing TGF-β1**.

**Figure 5.**
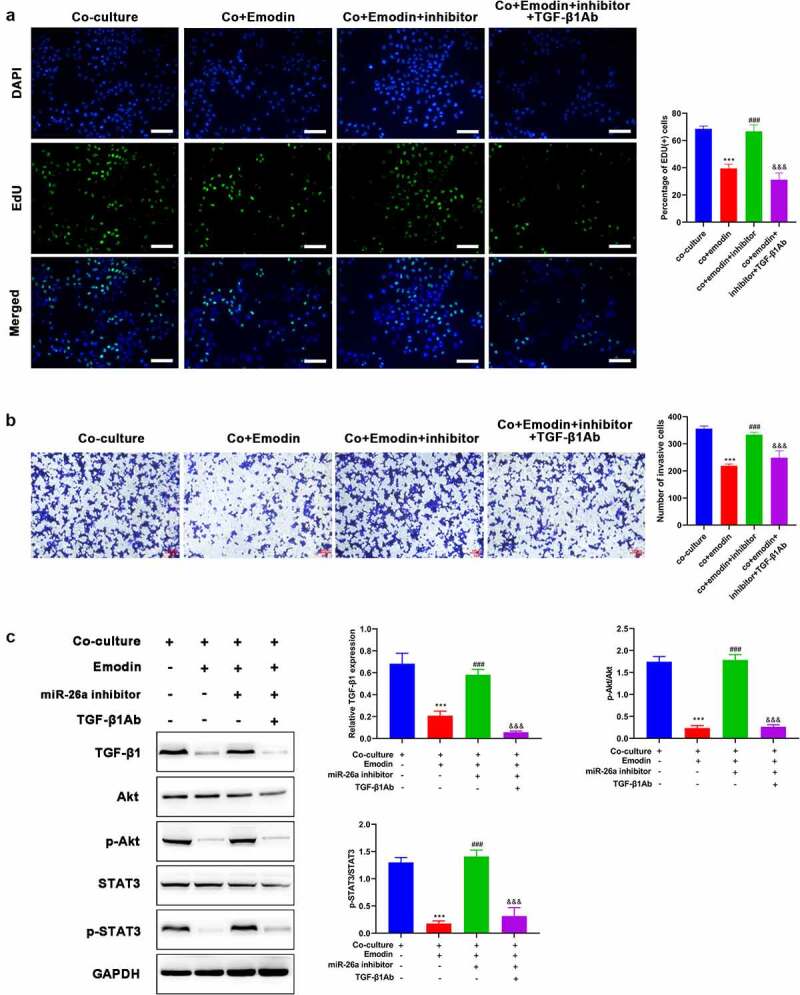
Down-regulated miR-26a blocks the effect of emodin on the proliferation and invasion of HepG2 cells from co-culture system by suppressing TGF-β1 and Akt activation. The co-culture systems formed by HepG2 cells and M2 macrophages were divided into four groups with different treatments (co-culture group: HepG2 cells were co-cultured with M2 macrophages without any treatment; co+Emodin group: under the treatment of emodin, HepG2 cells were co-cultured with M2 macrophages; co+Emodin+inhibitor group: under the treatment of emodin, HepG2 cells were co-cultured with M2 macrophages transfected by miR-26a inhibitors; co+Emodin+inhibitor+ TGF-β1Ab group: identical treatment to the co+Emodin+inhibitor group except a pretreatment of TGF-β1Ab. (a) The proliferation and (b) invasion of HepG2 cells were analyzed by Edu and transwell assays, respectively. (c) The protein levels of TGF-β1, Akt, and phosphorylated Akt were analyzed by Western blot. ***P < 0.005, vs. co-culture group; ^###^P < 0.005, vs. co+emodin group; ^&&&^P < 0.005, vs. co+emodin+inhibitor group. Scale bar = 100 μm.

To investigate the involvement of miR-26a/TGF-β1 axis in the suppression effect of emodin on the proliferation and invasion of HepG2 cells from the co-culture system, we next analyzed proliferation and invasion of HepG2 cells from co-culture system after M2 macrophages were transfected miR-26a inhibitors. As expected, miR-26a inhibitors reversed the effect of emodin on both the proliferation and invasion of HepG2 cells, but this effect could be blocked by the pre-treatment of TGF-β1Ab (Figure 5(a, b)). Given that Akt signaling is a critical pathway in multiple biological process including proliferation and invasion, the expression and phosphorylation of Akt were both detected from different groups. Western blot analysis hinted that emodin pretreatment before co-culture could reduce the expression of TGF-β1 and the phosphorylation of Akt, which could be reversed by miR-26a inhibitors (Figure 5(c)). Moreover, the addition of TGF-β1Ab blocked this function of miR-26a inhibitors (Figure 5(c)). These findings suggested miR-26a was involved in the effect of emodin on the cell proliferation and invasion of HepG2 from the co-culture system by suppressing TGF-β1/Akt signaling.

## Emodin suppresses tumor growth and promotes the polarization of M2 to M1 macrophages in HCC *in vivo*

**Figure 6.**
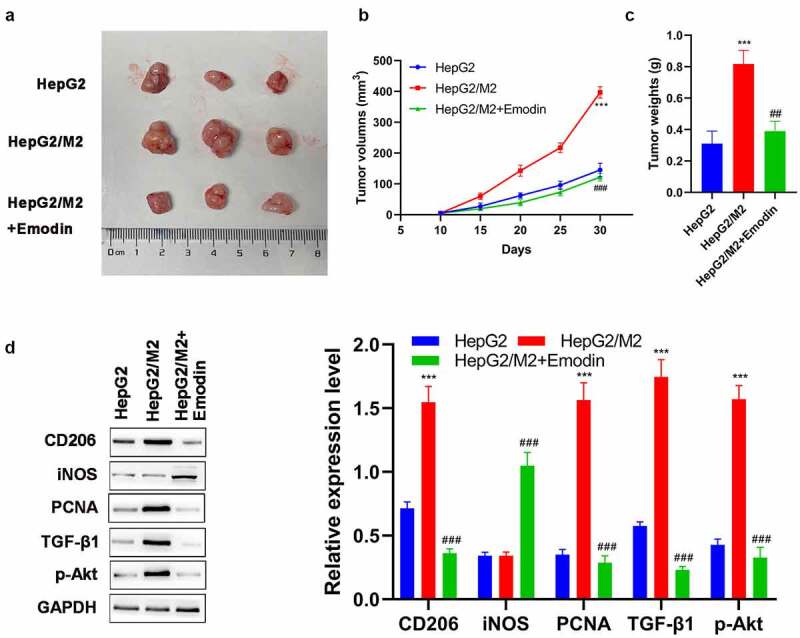
Emodin suppresses tumor growth and promotes the polarization of M2 to M1 macrophages in HCC *in vivo*. (a) The representative image, (b) growth curve, and (c) weight of xenografts collected from HepG2, HepG2/M2, and HepG2/M2+ emodin groups. (d) The protein expression of CD206, iNOS, PCNA, TGF-β1, and p-Akt in xenografts was detected by Western blot. ***P < 0.005, vs. HepG2 group; ^###^P < 0.005, vs. HepG2/M2 group.

To confirm the aforementioned results, we further performed *in vivo* analysis on BALB/c nude mice. Our results show that HepG2 cells promoted tumor growth after co-cultured with M2 macrophages (Figure 6(a,c)). On the other hand, this effect was effectively reversed by emodin treatment (Figure 6(a,c)). Then, the potential mechanism underlying the effect of emodin was investigated by Western blot. In the harvested xenografts, it was found that the expression of CD206, PCNA, and TGF-β1, as well as the phosphorylation of Akt (p-Akt) were significantly up-regulated after co-culture (Figure 6(d). Notably, compared with the HepG2/M2 group, additional emodin treatment (HepG2/M2+ emodin group) not only significantly reduced expressions of CD206, PCNA, TGF-β1, and p-Akt but also elevates the expression of iNOS. These data supported our *in vitro* analysis, indicating that emodin could reverse the promotion of growth in HepG2 induced by co-culturing with M2 macrophages via suppressing TGF-β1/Akt signaling.

## Discussion

In recent years, increasing literature highlights that TAMs contribute to the progression of diverse malignancies [37], including HCC [38]. M2 macrophages are the main phenotype of TAMs contributing to growth, invasion, and metastasis of tumor cells in the TME [39]. Therefore, M2 macrophages serve as potential therapeutic targets for cancer management. Moreover, M2 macrophage population has been implicated in the poor prognosis of patients with HCC [40]. In the present study, M2 macrophages induced by IL-4/IL-13 were used to investigate the role of TAMs in the proliferation and invasion of HepG2 and Huh7. Both IL-4 and IL-13 are common inducers of M2 macrophage activation [41]. Besides, it also has been reported that TGF-β1 shows a similar effect to IL-4/IL-13 that induces M2-like functional phenotypes [42]. Our study showed that M2 macrophages secreted an abundance of TGF-β1 to promote proliferation and invasion of both HepG2 and Huh7 cells. At the same time, HepG2 and Huh7 cells after co-culture produced TGF-β1 to maintain M2 macrophages, thereby forming a positive feedback loop. The *in vivo* analysis also showed co-culture with M2 macrophages effectively promoted the tumor formation of HepG2 and simultaneously up-regulated TGF-β1 expression. Emodin is a well-studied anti-tumor agent found in multiple Chinese medicinal herbs, which is potentially used for the treatment of breast cancer by suppressing the recruitment and M2 polarization of macrophages in tumors [43]. In this study, emodin induced the transformation of M2 to M1 macrophages and decreased TGF-β1 expression and Akt phosphorylation, thus breaking the vicious circle mediated by co-culture of HepG2 cells with M2 macrophages.

Consistent with previous reports [44,45], TGF-β1 treatment enhanced both the proliferation and invasion of HepG2 cells. Intriguingly, a similar result was observed in HepG2 cells after co-culture with M2 macrophages. In addition, we also found that the addition of TGF-β1Ab in a co-culture medium for neutralizing TGF-β1 could block the effect of M2 macrophages on both the proliferation and invasion of HepG2 and Huh7 cells, indicating that TGF-β1 might be the main contributor for the role of M2 macrophages in tumors.

Emodin can regulate macrophages toward M1 or M2 polarization based on specific conditions [16]. Our study, for the first time, demonstrated the intervention effect of emodin on the interaction between HepG2 cells and M2 macrophages. In the co-culture system, emodin treatment caused a reversion of M2 polarization in a concentration-dependent way, as confirmed by the decreased number of CD206+ cells and the increased number of CD86+ cells, as well as a shift of phenotypic or functional markers, such as Arg1, TGF-β1, and IL-10, toward an M1 polarization state (iNOS and TNF-α). Moreover, emodin also blocked the enhanced proliferation and invasion of HepG2 cells induced by co-culture with M2 macrophages.

Given that increasing research on microRNAs (miRNAs) has proved that the polarization of M1/M2 macrophage phenotypes could be regulated at transcription and post-transcriptional levels [46], we further investigated whether a miRNA is involved in the role of emodin in the interaction between HepG2 cells and M2 macrophages. Our study revealed that emodin up-regulated miR-26a expression in a concentration-dependent way. Meanwhile, silencing miR-26a in macrophages effectively blocked the suppressive effect of emodin on the M2 polarization of macrophages. 47,revealed that the down-regulation of miR-26a is related to the unfavorable prognosis of patients with HCC. 35,reported that miR-26a could repress the recruitment of macrophages in HCC. Based on bioinformatics analysis, our study further demonstrated that miR-26a directly targeted TGF-β1, and negatively regulated the expression of TGF-β1. Knockdown of miR-26a in M2 macrophages reversed the effect of emodin on the proliferation and invasion of HepG2 in the co-culture system, confirming the essential role of miR-26a. Notably, whether repressing miR-26a expression or not, the treatment of emodin led to the decrease of Arg1 and IL-10 and the increase of iNOS and TNF-α, indicating the polarization of M2 to M1 macrophages induced by emodin was not merely attributed to miR-26a. It was speculated that that is because of the multi-target property of emodin during treatment [48]. Emodin is related to Akt pathway in various diseases [49]. Akt pathway has been implicated in inflammatory signals, which can modulate M1/M2 polarization of macrophages [50]. The activation of Akt is required for M2 polarization [51,52]. Moreover, Akt activation is also involved in the pro-proliferation function of TGF-β1 [53]. Interestingly, the regulatory role of emodin in TGF-β1 expression and Akt phosphorylation was also reversed by miR-26a inhibitors. More importantly, the addition of TGF-β1Ab partly reversed the effect of miR-26a inhibitors. These data collectively revealed that emodin could suppress the enhanced proliferation and invasion of HepG2 mediated by co-culture with M2 macrophages through driving M2 macrophages to M1 polarization via miR-26a/TGF-β1/Akt. Finally, *in vivo* analysis confirmed that emodin could effectively diminish tumor formation ability of HepG2 induced by M2 macrophages, which was consistent with the results of *in vitro* analysis. Emodin also reduced the expression of PCNA, TGF-β1, and p-Akt in xenografts. Furthermore, higher expression of CD206 and lower expression of iNOS were observed in xenografts after HepG2 cells were co-cultured with M2 macrophages; then, emodin could interference with this effect of M2 macrophages by the repolarization of M2 macrophages via suppressing TGF-β1 and p-Akt.

There are some limitations in this study. For example, further studies involving other HCC cell lines are required to support our findings. Besides, the present study only investigated the role of TGF-β1 secreted by M2 macrophages in the proliferation and invasion of HCC cells, and the function of TGF-β1 secreted by HCC cells remains unknown, which is a direction of our future study. Moreover, M1 macrophages were not set as a negative control group in the current study, as our study focused on the crosstalk between M2 macrophages and HCC.

## Conclusion

Based on our data, M2 macrophages facilitated the proliferation and invasion of HepG2 cells by increasing the production of TGF-β1. Emodin could reverse these effects by driving M2 macrophage to M1 polarization via miR-26a/TGF-β1/Akt. Our study provides novel insights for a comprehensive understanding of the anti-cancer effect of emodin in HCC, and suggests the miR-26a/TGF-β1/Akt signaling axis might be a promising approach to modulate the TME in HCC.
